# Experiences of psychological mistreatment in older adults and promising practices: A scoping review protocol

**DOI:** 10.1371/journal.pone.0338374

**Published:** 2025-12-10

**Authors:** Sabrina Lessard, Annie Bernatchez, Houda Garrach, Mélanie Couture, Claire Godard-Sebillotte, Sarita Israel, Rym Zakaria

**Affiliations:** 1 Centre for Research and Expertise in Social Gerontology, CIUSSS West-Central Montreal, Montreal, Quebec, Canada; 2 Department of Anthropology, Université de Montréal, Montreal, Quebec, Canada; 3 School of Sociological and Anthropological Studies, University of Ottawa, Ottawa, Ontario, Canada; 4 Department of Nursing Sciences, Université du Québec à Trois-Rivières, Trois-Rivières, Quebec, Canada; 5 Research Chair on Mistreatment of Older Adults, Sherbrooke, Quebec, Canada; 6 School of Social Work, Université de Sherbrooke, Sherbrooke, Quebec, Canada; 7 Centre for Outcomes Research and Evaluation, Montreal, Quebec, Canada; 8 Department of Medicine, McGill University, Montreal, Quebec, Canada; UofM: The University of Memphis, UNITED STATES OF AMERICA

## Abstract

**Objective:**

This project aims to gain a thoroughly understanding of the characteristics and experiences of psychological mistreatment among older adults, acknowledging the diversity within this population. It also seeks to identify clinical tools and practices for its detection and intervention. While there is extensive literature on mistreatment of older adults, specific studies focusing on psychological aspects and intersecting social and identity dimensions are scarce. The findings will provide valuable insights for policymakers and healthcare professionals, helping to shape interventions and policies aimed at countering mistreatment in the ageing population.

**Introduction:**

Psychological mistreatment involves a range of behaviors, expressions, and gestures—or the lack of appropriate actions—that negatively impact an individual's health and dignity. Often subtle and difficult to detect, this type of mistreatment is prevalent and can coexist with other types of abuse. Examination of psychological mistreatment, shaped by various social and identity dimensions, is lacking in current research, particularly regarding how it is experienced by older adults. This scoping review seeks to map the current knowledge on psychological mistreatment of older adults, while highlighting gaps and future directions for research.

**Inclusion criteria:**

This scoping review will encompass studies that explore the characteristics and experiences of psychological mistreatment among older adults, including their experiences and those of perpetrators and witnesses. It will also identify clinical tools and practices for the detection and intervention of psychological mistreatment in this population.

**Method:**

A scoping review will be undertaken by a multidisciplinary team, examining studies from post-2010, sourced from both bibliographic databases and grey literature, available in English or French. Employing an intersectional framework, the review will use Gender-Based Analysis Plus (GBA+) to examine how different forms of discrimination intersect and shape experiences of mistreatment. That is, this approach will help explore how social and identity dimensions—including gender, age, sexual orientation, ethnicity, socioeconomic status, and health conditions—shape the experiences and manifestations of psychological mistreatment.

## Introduction

This project is part of a specific directive of the *ministère de la Santé et des Services sociaux* (MSSS) articulated in the Governmental Action Plan to Counter Mistreatment of Older Adults 2022–2027 (author's translation), sanctioned by the Quebec, Canada, government. It specifically corresponds to Orientation 5 of the Action Plan dedicated to “developing and disseminating knowledge relating to mistreatment” [1] (author's translation). This project also addresses Objective 5.1 [1], which involves generating new insights to enhance understanding of the forms of psychological mistreatment—whether through violence or neglect—as well as its characteristics, experiences, and manifestations, for both presumed victims and perpetrators. Consequently, this project aims to develop a foundational understanding of the characteristics, and experiences of psychological mistreatment of older adults, taking into account the diversity within this population. Another objective is to identify tools and clinical approaches for the detection of, and intervention in, psychological mistreatment.

Mistreatment of older adults is currently recognized as a significant public health concern and is internationally acknowledged as a violation of human rights [1–6]. Such mistreatment predominantly occurs within familial settings [7–11] and has important consequences on the health outcomes and life trajectories of older adults [12–14]. The complexity of mistreatment is extensive, encompassing multiple facets, and necessitates a comprehensive understanding that considers historical, cultural, and sociopolitical dimensions [15–16].

The definitions and prevalence of mistreatment have been shaped by sociocultural and moral shifts over the years, which have significantly influenced its perception, experiential reality, and theorization [17]. The recent decades have fostered the development of a specialized field focused on older adult mistreatment. This field has become increasingly structured, leading to a growing consensus, particularly within Quebec about the definitions and recognition of diverse types of mistreatment, including physical, psychological, financial and material, sexual, organizational, ageism, and those constituting violations of human rights [1,18].

In Quebec, psychological mistreatment is defined as “[a]ttitudes, words, gestures, or absence of appropriate actions that negatively affect an individual's psychological well-being or integrity [18].” Psychological mistreatment represents the most common, yet often the least observable types of mistreatment [18]. This type of mistreatment manifests in two primary forms: violence and neglect. Psychological violence encompasses behaviours such as “[e]motional blackmail, manipulation, humiliation, insults, infantilization, belittlement, verbal and non-verbal threats, disempowerment, excessive monitoring of activities, comments that are xenophobic, ableist, sexist, homophobic, biphobic, or transphobic [18].” Psychological neglect involves “[r]ejection, indifference, social isolation, disinterest, insensitivity, etc. [18]” This type of mistreatment typically occurs within relational dynamics, predominantly in adult child-parent interactions [19–21]: however, its implications extend beyond the direct victim-perpetrator relationship and are shaped by multiple factors. Moreover, psychological mistreatment frequently coexists with other types of mistreatment, and is often intensified by concurrent direct discrimination, such as racism, classism, ableism, heterosexism, cisgenderism, and ageism and can be perpetrated by a broader range of individuals.

The Survey on Elder Abuse in Quebec 2019 (SEAQ), conducted by Gingras [20], revealed that, according to the General Social Survey conducted by Statistics Canada in 1999, the prevalence of psychological mistreatment among individuals aged 65 and over was 7% (as cited in [20]) [22]. However, findings from the National Initiative for the Care of the Elderly in Canada (2016) indicated a prevalence rate of 2.7% [20]. More contemporary data from SEAQ reveals a self-reported prevalence of 4.6% for psychological mistreatment among adults aged 65 and over. Notably, a significant sex-based disparity exists, with women experiencing psychological mistreatment at a rate of 5.7% compared to 3.3% for men. Additionally, The Mistreatment Helpline, a provincial resource offering consultation, referral, and support by specialists on elder mistreatment and adults in vulnerable situations (lignemaltraitance.ca/en), reported that 30.8% of calls from April 2022 to March 2023 pertained to issues of psychological violence (26.4%) or psychological neglect (4.4%). These findings align with those of an international study by Yon et al. [23], which estimated that one in six adults over the age of 60 experiences some type of mistreatment, with approximately 12% reporting psychological mistreatment. Nonetheless, these statistics should be interpreted with caution as they likely represent only a fraction of the actual cases, do not account for this type of mistreatment against older adults in health care settings. Psychological mistreatment is often normalized, trivialized, or passively overlooked, which contributes to both underreporting and under-detection.

Numerous research studies and literature reviews have been devoted to various aspects of the mistreatment of older adults, encompassing the prevalence and characteristics of mistreatment [24], risk and protective factors [25], specific populations such as older women [23] and older members of ethnic minority groups [26], as well as adults with major neurocognitive disorders [27]. Considerable attention has also been paid to interventions [28–34], prevention strategies [35–39], and the development of detection tools [40–43]. Despite this extensive body of work, to the best of our knowledge, no literature review has specifically examined psychological mistreatment of older adults using an intersectional framework.

Moreover, it has been established that the experiences, perceptions, and acts of mistreatment toward older adults are influenced by a constellation of factors including cultural beliefs, values, norms, personal experiences, individual sensitivities, and the specific circumstances of those who are mistreated, those who perpetrate mistreatment, and those who witness it. Research demonstrates that even in cultures where filial piety is a core value, psychological mistreatment along with other types of mistreatment, can still occur among this population [44–45]. Further, investigations reveal that while there is a broad agreement on what constitutes mistreatment, individual interpretations of these incidents differ across gender, age, generation, and ethnic affiliations [46–47].

Researchers have pinpointed both individual traits—such as personality, substance consumption, financial reliance, and psychological disorders—and social factors—such as familial and intimate relationships, as well as residential conditions—as characteristics of those who commit mistreatment toward older adults, underscoring their diversity [48–53].

Research has also demonstrated how gender dimensions, interwoven with cultural and economic factors, contribute to instances of mistreatment, including psychological mistreatment [45,54]. Furthermore, studies from Quebec have elucidated both the immediate and enduring consequences of psychological mistreatment on the health and well-being of adults with disabilities [55]. Bédard et al. [56] have documented the psychological mistreatment faced by Lesbian, Gay, Bisexual (LGB) older adults, particularly through heterosexist practices and homophobic comments within shared living environments. Moreover, LGB older adults are potentially more vulnerable to mistreatment owing to factors such as isolation, past traumas, and a hesitancy to seek both formal and informal assistance [57–59]. Ultimately, these social and identity dimensions can affect the approaches adopted by professionals [60–62], volunteers [63], and family members [64] in their intervention with older adults.

A 2023 edition of the journal *Gérontologie et société* [*Gerontology and Society*] emphasizes the importance of acknowledging the diverse cultural and social contexts in which mistreatment is either partially expressed or remains unspoken [15]. This issue reveals, among other things, that psychological mistreatment is a societal phenomenon observable across diverse settings, embedded within routine social interactions among various populations. Consequently, older adults do not constitute a homogeneous group. Instead, like other stages of life, their experiences are characterized by distinct paths influenced by multiple life events that define everyone.

### Theoretical perspective

We claim that social and identity dimensions such as gender, age, sexual orientation, ethnic affiliation, socioeconomic status, and the presence of illness or disability, among others, are not immutable traits. Rather, these are constructs formed through diverse life circumstances and are intertwined with a spectrum of power dynamics, coexisting with multiple norms and values [65]. Such social and identity dimensions mirror the heterogeneity of individuals within society [66]. This heterogeneity is subject to evolution driven by global mobility and societal transformations marked by the individualization of identities, life choices, and the diversification of values and social roles [67]. Diversity transcends any definitive reference frameworks, applicable neither exclusively to migrants nor to non-migrants, nor confined to any specific minority or majority group. Indeed, these frameworks often emerge as complex. Consequently, diversity represents intricate and multilayered connotations.

This diversity extends to healthcare professionals who address mistreatment involving older adults as victims and adult persons as perpetrators. There are numerous ways to comprehend the world [68–70], interpret lived experiences, interpersonal relationships, and practices related to the detection and intervention of psychological mistreatment. These insights advocate for the application of the Gender-Based Analysis Plus (GBA+) framework [71], which integrate both gender-based and intersectional perspectives to account for the diverse situations, experiences, and perceptions where multiple forms of discrimination or oppression intersect—based on social and identity dimensions like gender, age, sexual orientation, ethnic affiliation, socioeconomic status, and the presence of illness or disability. The concept of intersectionality, first articulated by African American, Hispanic American, and Indigenous feminists [72] and theorized by Crenshaw [73], laid the groundwork for the development and later implementation of the GBA+ framework in public policy (introduced in 2005 and revised in 2011) in Canada and beyond [74]. GBA+ provides an analytical framework to comprehend and elucidate the complexity of human experiences [75]. This analytical tool acknowledges that perceived group affiliations subject individuals to diverse forms of prejudice and discrimination, influencing social interactions. It also emphasizes the importance of recognizing structural and systemic inequalities that disadvantage certain groups and hinder their development [76].

In this project, employing GBA+ will facilitate a deeper comprehension of psychological mistreatment by highlighting the distinct experiences of both older victims and perpetrators. This approach will also help to uncover how social and identity dimensions shape the experiences and manifestations of psychological mistreatment (e.g., do men and women experience psychological mistreatment differently, what are the diverse manifestations of psychological mistreatment among older LGB persons)? This approach will also allow the identification of detection and intervention tools that practitioners currently use when working with older adults from diverse social and cultural backgrounds. The objective is to cultivate a more detailed understanding of both the social and individual dynamics that contribute to psychological mistreatment, and to strengthen public policies to broaden the range of responses to mistreatment of older adults. The use of GBA + is particularly fitting for analyzing complex social phenomena, enabling a more integrated approach to the development of prevention, detection, and intervention programs [6]. This approach not only assists in crafting more inclusive and equitable public policies but also positions GBA+ as both an analytical framework and a practice committed to social justice.

## Materials and methods

We posit that psychological mistreatment is neither experienced nor perceived uniformly across different social and identity dimensions of those involved—whether as victims, perpetrators, or witnesses. Furthermore, the nature of psychological mistreatment and responses to it are contingent upon these social and identity dimensions. To examine the literature on psychological mistreatment, we consider the scoping review method to be best suited for providing a comprehensive overview of this phenomenon. A scoping review is particularly valuable because it incorporates a variety of data types beyond empirical research [77], including expert reports and grey literature. It is well-suited to broad research questions, allowing for the identification and characterization of studies available on a given topic. Its flexibility and iterative nature make it an ideal approach for exploring, mapping, and discussing tools and practices that support interventions [77].

This project does not involve formalized ethical approval as it is a scoping review, which is based on available scientific articles and publicly available reports or clinical tools. However, the authors will adopt ethical principles such as protecting confidentiality and mitigating harm by anonymizing all references to any places and people.

### Status and timeline of the study

The study is currently in the process of selecting relevant studies, which is expected to be completed by March 2025. The entire scoping review is anticipated to be finalized by September 2025.

The scoping review will be executed in six stages, adhering to the framework outlined by Levac et al. [78]. These stages are: (1) identifying the research question; (2) identifying relevant studies; (3) study selection; (4) charting the data; (5) collating, summarizing, and reporting the results; and (6) consultation.

An advisory committee, composed of co-researchers, collaborators, research professionals, students, and potential users of the results—including a representative from the user committee of the CIUSSS West-Central Montreal and one from The Mistreatment Helpline—is engaged at various stages of the process. Furthermore, members of the committee have collaboratively developed the scoping review protocol and contributed to the formulation of stages 1 and 2. They will renew their involvement for stages 5 and 6. Government partners have also been engaged in stages 1 and 2 and are slated for re-engagement during stages 5 and 6.

This protocol adheres to the PRISMA-P guidelines (see S1 Checklist), which stand for Preferred Reporting Items for Systematic Reviews and Meta-Analyses specific to scoping reviews. As the scoping review process is iterative, this protocol will serve as a foundational document. This ensures transparency and allows for the refinement of review processes over time.

#### Stage 1: Identify the research question.

The scoping review is structured around the following central question: How are the social and identity dimensions—such as age, gender, sexual orientation, ethnic affiliation, socioeconomic status, and the presence of diseases or disabilities in older adults—related to the characteristics of psychological mistreatment (objective 1), the manner in which it is experienced (objective 2), and what are the existing tools and clinical practices for the detection and intervention in cases of psychological mistreatment (objective 3)?

This scoping review is designed to:

**1. Objective 1a**: Identify the characteristics of psychological mistreatment, including indicators, risk factors, and protective factors, in:
  A. Older adults who experience psychological mistreatment;
  B. Individuals who perpetrate psychological mistreatment.
**2. Objective 1b**: Examine the impact of social and identity dimensions, such as gender, sexual orientation, ethnic affiliation, socioeconomic status, and the presence of diseases (i.e., mental health conditions, dependency, major neurocognitive disorders) or disabilities, on the characteristics of psychological mistreatment.
**3. Objective 2**: Explore the lived experiences (i.e., its manifestations) of psychological mistreatment according to the social and identity dimensions of:
  A. The older adult experiencing psychological mistreatment;
  B. The individual perpetrating psychological mistreatment;
  C. The individual witnessing psychological mistreatment.
**4. Objective 3**: Identify the existing tools and clinical practices for detection and intervention in the context of psychological mistreatment that are promising, both in Quebec and internationally.

#### Stage 2: Identify relevant studies.

In our pursuit of a comprehensive understanding of psychological mistreatment relative to social and identity dimensions, we have elected to focus this scoping review on two primary categories: the concept of psychological mistreatment and the population involved, which includes older adults, perpetrators, and witnesses. This focused approach enables us to incorporate all relevant social and identity dimensions and to cover various contexts where such mistreatment might occur, including home, community, and institutional settings. This strategic decision is intended to ensure a comprehensive exploration of the phenomena across diverse environments.

***Inclusion and exclusion criteria*:** Publications that satisfy the established criteria related to the concept of psychological mistreatment and the populations under study—including older adults, perpetrators, and witnesses—will be included in the scoping review. This inclusion will ensure a comprehensive assessment of the available literature pertinent to our research objectives.

***Concept*:** The central concept of this scoping review is defined as psychological mistreatment, drawing on definitions provided by leading sources such as the Research Chair on Mistreatment of Older Adults and the Centre for Research and Expertise in Social Gerontology as described at the beginning of the article.

We also consider that psychological mistreatment may encompass direct discriminatory practices, defined as unfavorable treatment toward individuals belonging to specific social groups [79]. Such discrimination may be based on factors such as skin color, ethnic or national origin, presence of illness or disability, sex, gender, sexual orientation, and/or socioeconomic status, and it can manifest through xenophobic, ableist, sexist, homophobic, biphobic, transphobic, and ageist remarks.

In this scoping review, psychological mistreatment (violence, neglect and discrimination) must be experienced directly and individually within a trust-based relationship—whether with an individual, a community, or an organization where trust is expected—and can unintentionally or deliberately cause harm or distress to an adult, thereby constituting a breach of their well-being or psychological integrity.

If the article addresses several types of mistreatment, to be included, the results must focus primarily (i.e., more than 50% of the findings) on psychological mistreatment (violence, neglect, and discrimination).

***Population*:** We will examine three distinct categories of populations. The primary population comprises individuals subjected to psychological mistreatment, specifically adults aged 65 and above. The secondary population encompasses those who perpetrate psychological mistreatment or discrimination against older adults. The third population refers to individuals who observe instances of such mistreatment, including healthcare providers and social service professionals.

1. The study sample predominantly consists of adults aged 65 and older who experience psychological mistreatment;
2. Individuals of all ages who engage in perpetrating psychological mistreatment;
3. Individuals of all ages who witness psychological mistreatment.

Our primary interest lies in the literature focusing on these populations and examining the social and identity dimensions discussed above.

***Type of literature*:** Considering that research on psychological mistreatment of older adults has gradually advanced over recent decades, this scoping review will be restricted to studies published from 2010 onward, thus covering the past 15 years. It will include empirical studies (qualitative, quantitative and mixed methods), and exclude conference abstracts, editorials, commentaries, book summaries, blogs, and literature reviews—such as systematic reviews, meta-analyses, scoping reviews, and narrative reviews. Eligible studies must be written in either French or English.

#### Stage 3: Studing the selection.

***Sources of information*:** The literature will be identified through three categories of information sources: scientific databases, grey literature, and consultations with experts and collaborators specializing in the prevention of mistreatment toward older adults.

**1. Databases**: The review will encompass databases from both health sciences and social sciences, including some that are specifically focused on intersectional issues: Érudit, CAIRN, CINAHL, APA PsycInfo, MEDLINE, Gender Studies Database, Family Studies Abstracts, and Social Work Abstracts from EBSCO Information Services.
**2. Grey Literature**: Grey literature from Quebec and Canada will be examined. This includes, but is not limited to, community organization websites (e.g., DIRA-Estrie, DIRA-Laval, Carrefour Montrose, *Ressources ethnoculturelle contre l’abus envers les aînés*, *SOS Aînés Maltraitance*), government websites (e.g., *ministère de la Santé et des Services sociau*x of Quebec, Institut national de santé publique du Quebec (INSPQ), Canadian Network for the Prevention of Elder Abuse (CNPEA), and Wilson Center) and research center websites (e.g., Research Chair on Mistreatment of Older Adults). This category also encompasses expert reports.
**3. Experts and Collaborators**: Quebec experts on mistreatment toward older adults, along with our collaborators, will participate in an online workshop to identify unindexed literature and unpublished clinical tools and practices for detection and intervention, thereby enriching the search strategy. These experts comprise regional coordinators for the prevention of mistreatment of older adults, professionals from community organizations and health and social service institutions, as well as our collaborators, partners, and potential end users of our study’s findings.

***Research strategy*:** The librarian (Zakaria R.) at the Centre for Research and Expertise in Social Gerontology played a pivotal role in formulating the research strategy. She integrated the two primary concepts to initiate the database search. The refinement process for the research strategy was conducted collaboratively by the librarian and the authors over a three-month period, from March to May 2024, with inputs from our partners consulted in April 2024. The research strategy was initially developed for CINAHL-Complete (Ebsco) and subsequently adapted for each additional database. Keywords were searched within titles, abstracts, uncontrolled vocabulary keywords, and controlled vocabulary (indexed terms or terms derived from the database’s thesaurus). The concepts and keywords used in the database search are given in Table 1.

**Table 1:** Concepts and keywords used in databases.

CINAHL Complete (Ebsco) – December 2024	
Concept	Query
Psychological mistreatment	TI ((psychological or emotional) N2 (mistreatment or maltreatment or abuse or violence or manipulation or neglect or harassment or aggression)) OR AB ((psychological or emotional) N2 (mistreatment or maltreatment or abuse or violence or manipulation or neglect or harassment or aggression)) OR TI (Verbal N2 (mistreatment or maltreatment or abuse or violence or harassment or aggression)) OR AB (Verbal N2 (mistreatment or maltreatment or abuse or violence or harassment or aggression)) OR TI (Moral N2 (abuse or harassment)) OR AB (Moral N2 (abuse or harassment)) OR TI (spiritual N2 (mistreatment or abuse)) OR AB (spiritual N2 (mistreatment or abuse)) OR TI (intimidation or bullying or gaslighting or discrimination or infantilization or stigma* or coercive control) OR AB (intimidation or bullying or gaslighting or discrimination or infantilization or stigma* or coercive control) OR (MH “Emotional Abuse”) OR (MH “Verbal Abuse”) OR (MH “Bullying”) OR (MH “Discrimination”) OR (MM “Stigma)
	AND
Aged population	TI (Ag#ing or elder* or seniors) OR AB (Ag#ing or elder* or seniors) OR TI ((Late# life or late-life)) OR AB ((Late# life or late-life)) OR TI (Old* N2 (m?n or wom?n or people or population# or adult# or person#)) OR AB (Old* N2 (m?n or wom?n or people or population# or adult# or person#)) OR (MM “Aged, 80 and Over+”) OR (MM “Frail Elderly”) OR (MM “Aged+”) OR (MH “Aged, 80 and Over+”) OR (MH “Frail Elderly”)
Limits Date: 20100101–20241231 Expanders – Apply equivalent subjects Narrow by Language: – French Narrow by Language: – English Search modes – Boolean/Phrase	

***Grey literature*:** In the Google search engine, the following keywords were employed: psychological mistreatment, emotional mistreatment, spiritual mistreatment, and older adult or senior. Additionally, the websites of the previously mentioned organizations were examined.

***Data management*:** All documents will be imported into Covidence (Veritas Health Innovation Ltd., Melbourne, Australia), an online tool designed to streamline processes in scoping reviews, including literature management, duplicate removal, and independent document selection by multiple team members. Covidence also allows for the import of full texts to facilitate data extraction.

Grey literature will be catalogued in an Excel file, with entries noting the organization or website, date of selection, and type of publication.

***Selection process*:** Initially, the research team members will select 10% of the studies to standardize the selection process and refine the inclusion and exclusion criteria—team members will meet until a consensus is reached. Study selection will then proceed in two stages:

**1. Selection of Titles and Abstracts**: The selection of titles and abstracts will be conducted independently by two members of the research team (five members of the team were included at this stage). In the event of disagreements regarding the relevance of studies, the lead researcher will mediate the resolution. If unresolved conflicts persist, an additional meeting with all the project team members will be convened. Studies deemed ambiguous will be assigned a status of ‘uncertain.’ The inclusion or exclusion criteria for each study will be documented in the ‘note’ section of Covidence.
**2. Selection of Full Texts**: In the second phase of the selection process, the full texts will be independently reviewed by two members of the research team. An initial calibration process will be conducted on approximately ten studies to ensure consensus on the selection criteria. As in the previous stage, reasons for exclusion will be recorded. Study with uncertain inclusion will be discussed by the team members to determine whether they meet the selection criteria. Additionally, the references of the selected studies will be reviewed to identify any additional relevant studies.

The same process will be applied to grey literature, with the difference that it will be conducted using Excel.

#### Stage 4: Charting the data.

Using an extraction framework inspired by Noyes and Popay [80], data from each study will be descriptively extracted and analyzed, incorporating GBA + , addressing both social and identity dimensions. Data extraction will be conducted independently by two project team members and validated by a third member. Studies with ambiguity will be reviewed collaboratively by the team members. Data will be extracted from Covidence for studies and from Excel for grey literature, and will be organized according to the following categories:

***Empirical studie*s**:

**1. General Data**: Title, year of publication, authors’ names, field of study or discipline of the first author, country, and research objectives.
**2. Theoretical Framework**: Definition of psychological mistreatment, the theoretical approach guiding the research project, or the clinical tool and practice for detection and intervention.
**3. Methodology**: Context, samples, participant characteristics (i.e., number, age, gender, and other identity and social dimensions), methods of data collection and analysis, as well as the strengths and limitations identified by the authors.
**4. Results**:
  a. The characteristics of psychological mistreatment (i.e., indicators, risk factors, and protective factors):
    A. Among older adults experiencing psychological mistreatment;
    B. Among perpetrators of psychological mistreatment.
  b. The influence of social and identity dimensions, including gender, sexual orientation, ethnic affiliation, socioeconomic status, and the presence of illness such as mental health issues, dependency, and major neurocognitive disorders, or disabilities on the characteristics of psychological mistreatment.
    A. Among older adults experiencing psychological mistreatment;
    B. Among perpetrators of psychological mistreatment.
  c. Experiences of psychological mistreatment shaped by social and identity dimensions:
    A. From the perspective of the older adult undergoing psychological mistreatment;
    B. From the perspective of the individual perpetrating psychological mistreatment;
    C. From the perspective of the witness of psychological mistreatment.
  d. Clinical tools and practices for detection and intervention in psychological mistreatment of older adults: This encompasses the specific demographic intended for each instrument as well as recommendations for the enhancement of these tools and practices.

***Assessment of methodological quality*:** The evaluation of methodological quality is not required and will therefore be excluded. However, a thorough analysis of the data’s relevance will be undertaken. In the final phase of the project, we will seek feedback on the initial findings from our collaborating partners.

#### Stage 5: Collating, summarizing, and reporting the results.

A preliminary descriptive analysis will be conducted for each category of empirical studies. Subsequently, a transversal thematic analysis [81] will be conducted to synthesize prevailing understandings and to more precisely identify experiences of psychological mistreatment in relation to the social and identity dimensions of older adults. This thematic analysis will consider the research aims, especially focusing on psychological mistreatment within diverse contexts. A convening of project team members and collaborating partners will facilitate an iterative and reflective approach, steering this stage as necessary.

#### Stage 6: Consultation.

The concluding stage entails consultations with experts, knowledge users, older adults, caregivers, and users of health and social services. The objective of this stage is to unearth scientific publications, grey literature, tools, and clinical practices potentially overlooked in the literature review, such as specific intervention strategies. This stage also provides an opportunity to disseminate preliminary research findings. These consultations will manifest as an online workshop, facilitated by the Centre for Research and Expertise in Social Gerontology (creges.ca/en/) knowledge mobilization team. Partners will be encouraged to offer insights and to engage actively in the workshop.

## Discussion

This project holds the potential to yield impactful insights into the lesser-explored dimensions of psychological mistreatment against older adults, thus informing practitioners, managers, policymakers, and public policy frameworks. The project aims for outcomes that resonate significantly across various levels of application. Therefore, the results, expected by the *ministry*, are poised to be integrated into public policies and ministerial action plans. This integration is anticipated to contribute to the creation of clinical tools and the refinement of detection and intervention practices designed to counter psychological mistreatment of older adults.

The scoping review will aggregate and scrutinize a broad spectrum of existing empirical research, expert reports, and grey literature. The focus will be on delineating the characteristics of psychological mistreatment—including indicators, risk, vulnerability, and protective factors—and examining how such mistreatment is experienced by older adults, perpetrators and witnesses, with particular attention to social and identity dimensions. Furthermore, this project will aid in identifying tools and practices for detection and intervention while assessing the barriers and facilitators to their utilization.

The uniqueness of this project is attributed to the diverse composition of the team assembled to execute it. The team comprises researchers, clinicians, potential users of the results, older adults, and caregivers who are recipients of health and social services, alongside experts in elder mistreatment. This multidisciplinary collaboration enhances the breadth and depth of the project, ensuring a comprehensive approach to addressing the psychological mistreatment of older adults.

### Limitations of the study design

The main limitation of this protocol is the scope of the integrated grey literature, which primarily focuses on Quebec and Canada. Since this project is linked to a ministerial mandate and aims to benefit the entire province of Quebec, we have decided to emphasize regional initiatives. However, scientific articles, which have an international scope, are expected to offset this limitation.

### Dissemination plans

Without being limited to this, this scoping review will result in several scientific productions, including a two-page summary document aimed at practitioners and the general public, a research report, two open-access scientific articles (one in English and one in French), and presentations at different scientific conferences. The results will also be summarized in a policy brief for decision-makers, aimed at outlining the findings and providing guidance and recommendations to help to counter the phenomenon. The results may also inform recommendations included in a brief to support the development of the next action plan to fight against mistreatment.

### How amendments to the study, including termination, will be dealt with

Amendments to the study, including termination, will be managed through a structured process. Any proposed changes must be submitted and approved by the Advisory Committee. This ensures that all modifications will be thoroughly evaluated for their impact on the study’s objectives.

## Supporting information

S1 Checklist The PRISMA-ScR checklist is annexed. (DOCX)
